# Effect of chlorhexidine-containing prophylactic agent on the surface characterization and frictional resistance between orthodontic brackets and archwires: an in vitro study

**DOI:** 10.1186/2196-1042-14-48

**Published:** 2013-11-20

**Authors:** Tahereh Hosseinzadeh Nik, Tabassom Hooshmand, Habibeh Farazdaghi, Arash Mehrabi, Elham S Emadian Razavi

**Affiliations:** 1 Department of Orthodontics, Dental Research Center, School of Dentistry, Tehran University of Medical Sciences, Tehran 14174, Iran; 2 Department of Dental Biomaterials, School of Dentistry/Research Center for Science and Technology in Medicine, Tehran University of Medical Sciences, Tehran 14174, Iran; 3 Department of Oral and Maxillofacial Radiology, School of Dentistry, Khorasgan (Isfahan branch), Islamic Azad University, Isfahan 16578, Iran

**Keywords:** Bracket, Wire, Physical property, SEM, Friction, Chlorhexidine

## Abstract

**Background:**

The purpose of this study was to assess the surface characterization and frictional resistance between stainless steel brackets and two types of orthodontic wires made of stainless steel and nickel-titanium alloys after immersion in a chlorhexidine-containing prophylactic agent.

**Methods:**

Stainless steel orthodontic brackets with either stainless steel (SS) or heat-activated nickel-titanium (Ni-Ti) wires were immersed in a 0.2% chlorhexidine and an artificial saliva environment for 1.5 h. The frictional force was measured on a universal testing machine with a crosshead speed of 10 mm/min over a 5-mm of archwire. The surface morphology of bracket slots and surface roughness of archwires after immersion in chlorhexidine were also characterized using a scanning electron microscope (SEM) and an atomic force microscope (AFM), respectively.

**Results:**

There was no significant difference in the frictional resistance values between SS and Ni-Ti wires immersed in either chlorhexidine or artificial saliva. The frictional resistance values for the SS and Ni-Ti wires immersed in 0.2% chlorhexidine solution were not significantly different from that inartificial saliva. No significant difference in the average surface roughness for both wires before (as-received) and after immersion in either chlorhexidine or artificial saliva was observed.

**Conclusions:**

One-and-half-hour immersion in 0.2% chlorhexidine mouthrinse did not have significant influence on the archwires surface roughness or the frictional resistance between stainless steel orthodontic brackets and archwires made of SS and Ni-Ti. Based on these results, chlorhexidine-containing mouthrinses may be prescribed as non-destructive prophylactic agents on materials evaluated in the present study for orthodontic patients.

## Background

Orthodontic sliding mechanics is a technique used for closing space, usually achieved by moving brackets along the arch wire or sliding the wire through the brackets and the molar tubes. Friction is a major disadvantage affecting sliding mechanics and is generated by the contact between the bracket and the archwire [1]. Friction is a force that resists the relative motion of two objects in contact, and its direction is tangential to the shared interface of the surfaces [2]. Frictional resistance during sliding mechanics must be kept to a minimum in order that orthodontic tooth movement can be generated through light optimal forces [1].

Factors that may influence orthodontic frictional resistance include bracket and archwire materials, relative bracket-wire clearance, wire size, archwire section (round vs rectangular), torque at the bracket-wire interface, surface conditions of the archwires and bracket slot, and type and force of ligation [3]–[7]. An additional factor that may influence friction is saliva, but the application of prophylactic mouthwashes and dental hygiene products and their effects on orthodontic appliances have not been fully investigated.

Mouthwashes are clinically useful for reducing plaque accumulation during the active phase of orthodontic treatment [8]. However, their components may cause corrosion and discoloration of stainless steel and titanium alloys. Resistance to corrosion of stainless steel and titanium wires depends on the formation of a passive oxide layer. If this layer deteriorates, the arch wire may be exposed to corrosion [9,10]. Corrosion and its effect of increasing surface roughness have the potential to increase frictional force at the wire-bracket interface [11]. The effects of fluoride-containing products on the metal wires and bracket corrosion and frictional resistance have been evaluated, and the detrimental effect of fluoride ions has been reported [11]–[15]. Kao et al. [12] investigated the frictional resistance between metal brackets and different types of orthodontic wires after immersion in other prophylactic agents such as a fluoride-containing prophylactic solution (acidified phosphate fluoride (APF) agent). It was shown that static frictional resistance for the stainless steel, heat-activated nickel-titanium, and beta-titanium alloy wires immersed in 0.2% APF solution was significantly higher than that of those immersed in an artificial saliva. Watanabe and Watanabe [16] have also shown changes in the surface color and morphology of titanium-based orthodontic wires after immersion in an APF agent for 24 h. Similarly, Huang [11] reported that lower fluoride-containing (<2,500 ppm) environments had no appreciable influence on the surface roughness variation for Ni-Ti archwires after a 28-day immersion test. In high-fluoride (17,000 ppm), gel-containing artificial saliva, significant changes in corrosion morphology and surface roughness were observed. Several studies have revealed that fluoride ions can destroy the protective and passive TiO_2_ film on the Ti or Ti alloy surface, leading to a deterioration of corrosion morphology [10,17,18]. One possible explanation may be that high fluoride concentrations stay localized and attack the bracket-archwire interface. This increases the frictional force between the bracket and archwire commensurate with the increase in surface roughness. Therefore, the effectiveness of arch-guided tooth movement would decrease [11].

Antiseptic mouthrinses such as chlorhexidine may be prescribed for reducing plaque accumulation in the active phase of orthodontic treatment when mouth hygiene may be compromised [8,19]. In addition, chlorhexidine can reduce the severity of traumatic ulcers and gingivitis levels during orthodontic therapy [20].

To date, the effects of chlorhexidine mouthrinses on the frictional resistance between orthodontic brackets and wires have not been reported. Therefore, the purpose of this study was to assess the frictional resistance between stainless steel brackets and two types of orthodontic arch wires made of stainless steel and nickel-titanium alloys after immersion in a chlorhexidine-containing prophylactic agent. The surface morphology of bracket slots and surface roughness of arch wires after immersion in the chlorhexidine were also characterized using a scanning electron microscope (SEM) and an atomic force microscope (AFM), respectively.

## Methods

Forty upper premolar stainless-steel metal brackets (standard edgewise DentsplyGAC International, Islandia, NY, USA) with 0.022-in slot size were selected. Two types of orthodontic wires including 0.019 × 0.025-in standard rectangular stainless steel (3 M Unitek, Monrovia, CA) and 0.019 × 0.025-in heat-activated nickel-titanium (Ni-Ti, 3 M Unitek) wires were used. The ligation between the bracket and wire was an elastic module (o-ring, Dentaurum intraoral elastics, Dentaurum GmbH & Co. KG, Ispringen, Germany).

### Frictional resistance evaluation

The wires were cut into 5-cm-long specimens. The brackets and wires were cleaned with alcohol wipes before the module or ligatures were tied to form a test unit. All experimental units were immersed in 0.2% chlorhexidine mouthrinse (chlorhexidine gluconate, hydrogenated castor oil, sorbitol, and alcohol, Shahdaru Labratories, Tehran, Iran) at 37°C for 1.5 h. The control units were immersed in a modified Fusayama artificial saliva (NaCl 400 mg/L, KCl 400 mg/L, CaCl_2_ · 2H_2_O 795 mg/L, NaH_2_PO_4_ · H_2_O 690 mg/L, Na_2_S · 9H_2_O 5 mg/L, urea 1,000 mg/L, pH 6.75) at 37°C for 1.5 h. Each wire-bracket combination was immersed in an individual 15-mL plastic tube. Ten specimens of each wire-bracket combination were used in each group. The specimens were removed from their respective solutions and rinsed with distilled water. After drying, the wires were tied to the brackets with elastic modules.

**Figure 1:**
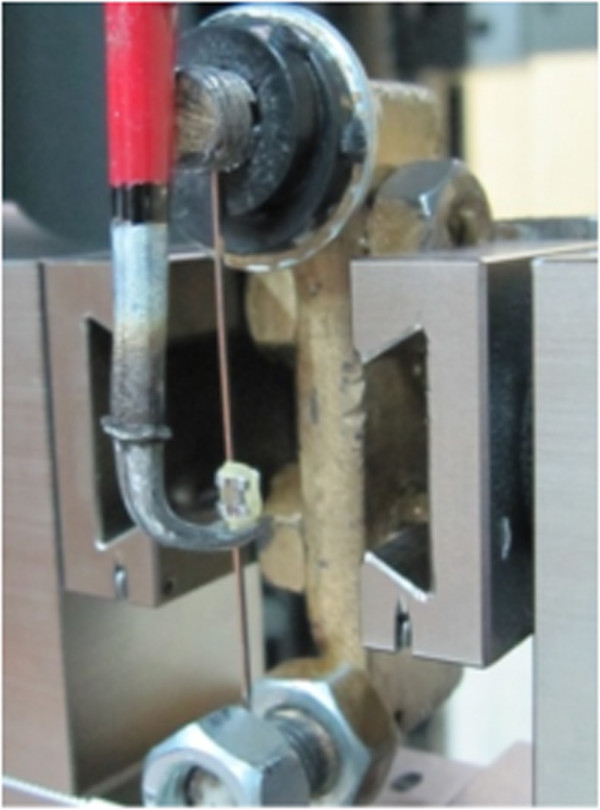
Friction testing apparatus.

Frictional force was measured using a universal testing machine (Zwick/Roell Z050, Germany). A custom-made fixture was designed for holding wires as shown in Figure 1. A plumb line was suspended to ensure that the bracket mount was parallel with the vertical line scribed on the steel bar base of the bracket mount assembly. A load cell was calibrated between 0 and 5 N, and the archwire was drawn through the bracket at a crosshead speed of 10 mm/min over a 5-mm section of archwire. Care was taken to avoid introducing torsion into the test specimen during clamping. Static friction was recorded as the maximum frictional force required to generate initial movement of the bracket over the 5-mm test distance. After each test, the bracket-wire combination was removed, and a new assembly was placed. The recorded data were analyzed using two-way analysis of variance (ANOVA) to determine significant differences between the two types of archwires immersed in two test environments. Statistical analysis was performed using statistical software (SPSS 16 for Windows; SPSS Inc., Chicago, IL, USA) at the 0.05 significance level.

### Surface characterization

All specimens were cleaned with 95% ethanol before SEM and AFM observations. Scanning electron micrographs of the slot surfaces of the as-received stainless steel metal brackets and after immersion in the 0.2% chlorhexidine mouthrinse and artificial saliva were recorded using a SEM (DSM 960A, Carl Zeiss AG, Germany).

The surface topography and roughness of the stainless steel (SS) and nickel-titanium (Ni-Ti) archwires before (as-received) and after immersion in the chlorhexidine and artificial saliva were evaluated using an AFM (scanning probe microscope Solver PRO Zelenograd, Moscow, Russia). Scanning was carried out in air and at a scanning rate of 10 Hz. New immersed specimens were used for the topography test. Surface roughness measurements were taken using three specimens per material, and two measurements over an area of 30 × 30-μm were generated per specimen. The recorded data of surface roughness before and after immersion test were statistically analyzed using two-way ANOVA. The Tukey test was applied for multiple comparison analysis with *P* < 0.05, indicating significant statistical difference.

## Results

Table 1 shows statistical analysis of the results of the surface roughness and friction resistance tests by ANOVA. The frictional resistance values of the stainless steel and nickel-titanium wires with the stainless steel brackets after immersion in the chlorhexidine and artificial saliva (control group) are presented in Table 2.

**Table 1:** Statistical analysis of the results of the surface roughness and friction resistance tests by ANOVA

	**Friction resistance**				**Surface roughness**			
	***df***	**Mean square**	***F***	**Significance**	***df***	**Mean square**	***F***	**Significance**
Archwire	1	0.067	1.588	0.215	1	3.799	0.037	0.850
Test environment	1	0.104	2.471	0.125	1	23.55	0.228	0.638
Interaction (archwire × environment)	1	0.032	0.763	0.388	1	145.81	1.41	0.249
Error	37	0.42			20	103.40		
Total	41				24			
Corrected total	40				23			

**Table 2:** **Frictional resistance values (N/mm**^**2**^**) for the nickel-titanium and stainless steel wires with stainless steel brackets**

	**Chlorhexidine**				**Artificial saliva**			
	**Mean**	**SD**	**Min**	**Max**	**Mean**	**SD**	**Min**	**Max**
Nickel-titanium archwire	0.387	0.189	0.212	0.761	0.331	0.214	0.161	0.532
Stainless steel archwire	0.412	0.219	0.071	0.742	0.367	0.169	0.183	0.711

*SD*, standard deviation.

Statistical analysis as shown in Table 1 revealed that the interaction of the two factors (type of wire and test environment) was not significant (*P* > 0.05). The mean frictional resistance values for both Ni-Ti and SS wires immersed in the chlorhexidine were higher than those recorded for wires immersed in artificial saliva, but these differences were not statistically significant (*P* > 0.05). In addition, no significant difference in the frictional resistance between the two types of wires in either of test environments was observed (*P* > 0.05).

**Figure 2:**
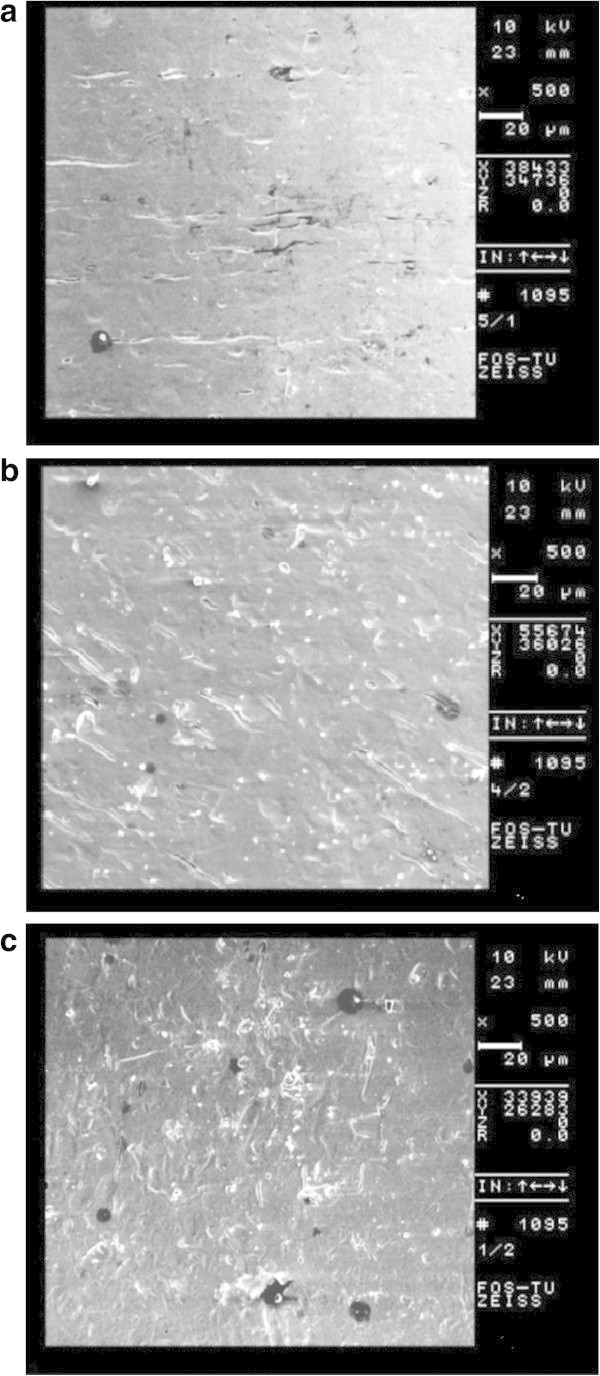
**SEM photomicrographs of stainless steel bracket slots. (a)** As-received bracket, **(b)** after 1.5-h immersion in the artificial saliva, and **(c)** after 1.5-h immersion in chlorhexidine mouthrinse. Magnification is × 500.

SEM photomicrographs of the as-received stainless steel bracket slots and after 1.5-h immersion in the artificial saliva and chlorhexidine mouthrinse are presented in Figure 2a,b,c. From the SEM images, it was evident that the slot surface of brackets immersed in the two test environments was slightly more porous than that of the as-received brackets.

**Figure 3:**
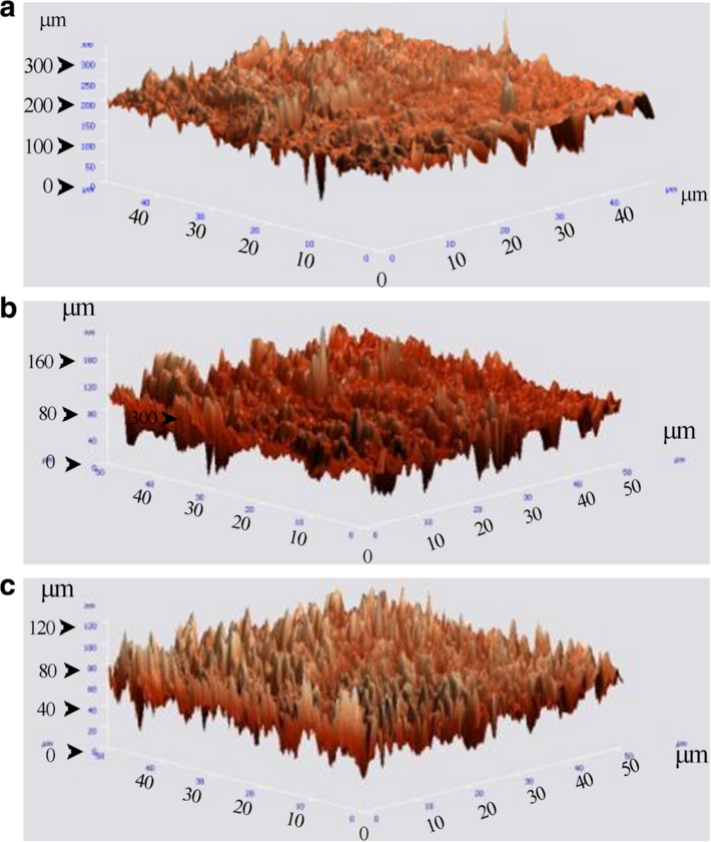
**AFM observations of the SS archwire. (a)** As-received SS wire, **(b)** after 1.5 h immersion in the artificial saliva, **(c)** after 1.5-h immersion in the chlorhexidine mouthrinse. Scanning area is 30 × 30 μm.

**Figure 4:**
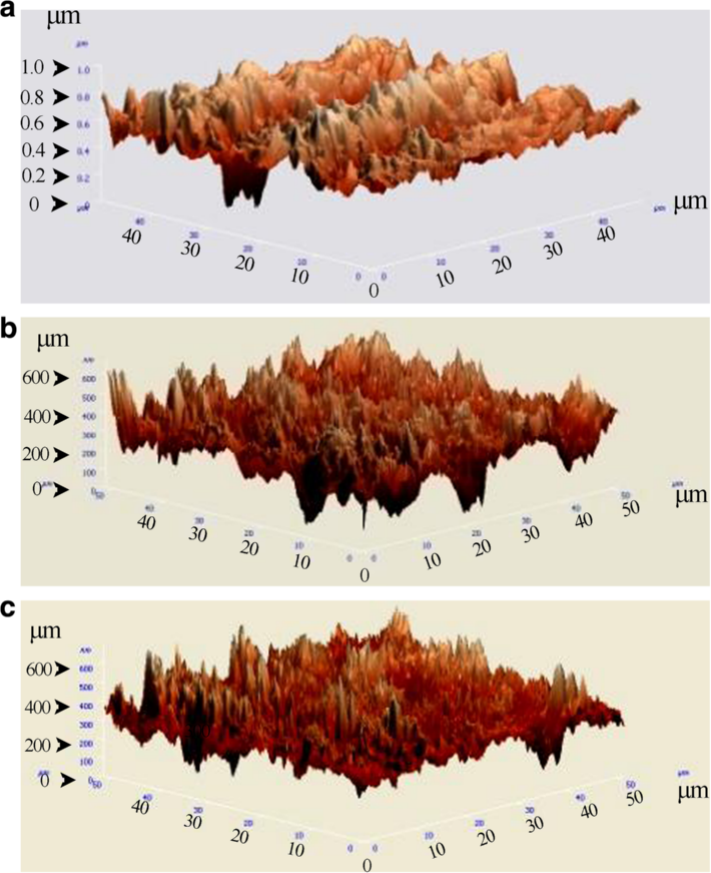
**AFM observations of the Ni-Ti archwire. (a)** As-received Ni-Ti archwire, **(b)** after 1.5-h immersion in the artificial saliva, **(c)** after 1.5-h immersion in the chlorhexidine mouthrinse. Scanning area is 30 × 30 μm.

Figures 3 and 4 represent the AFM observations of the as-received SS and Ni-Ti archwires, and after their immersion in the chlorhexidine and artificial saliva test environments, respectively. No significant difference in surface morphology was observed for either wires before and after immersion in either of test environments.

Table 3 shows the surface roughness values obtained by AFM for the as-received SS and Ni-Ti archwires and after immersion in the chlorhexidine and artificial saliva test environments. The average roughness value for the as-received Ni-Ti was significantly higher than that of the SS wire (*P* < 0.05), which was also confirmed by AFM observation. From the results, immersion in the chlorhexidine or artificial saliva environments had no significant influence on the average surface roughness values for the two archwires when compared with that of the as-received wires (*P* > 0.05). In addition, there was no significant difference between the surface roughness for each type of wire immersed in chlorhexidine and that of those immersed in the artificial saliva (*P* > 0.05).

**Table 3:** Surface roughness values (μm) for the as-received wires and after immersion in chlorhexidine and artificial saliva

	**As-received**				**Chlorhexidine**				**Artificial saliva**			
	**Mean**	**SD**	**Min**	**Max**	**Mean**	**SD**	**Min**	**Max**	**Mean**	**SD**	**Min**	**Max**
Nickel-titanium	67.95	44.30	62.8	73.1	64.82	39.85	59.68	69.98	67.77	36.79	62.63	72.93
Stainless steel	10.74	3.20	5.59	15.89	9.27	3.45	4.12	14.42	10.35	2.75	5.21	15.51

*SD*, standard deviation.

## Discussion

The effect of chlorhexidine prophylactic agent on the frictional resistance between orthodontic metal brackets and archwires has not been previously investigated. Because of the influence of many factors, defining exact amount of friction generated during orthodontic treatment is difficult. The influencing factors are due to the type of bracket and wire, and due to the different closing space mechanics employed.

In the present study, the effect of chlorhexidine on the frictional resistance of stainless steel brackets and two types of orthodontic wires made of stainless steel and nickel-titanium alloys was investigated. Static friction was evaluated because the sliding movement of teeth along an arch wire is not continuous, but occurs in a series of short steps or jumps. Therefore, static friction is considered to be more important than kinetic friction because it needs to be overcome each time the tooth moves [21].

The results showed that frictional resistance values for immersed Ni-Ti and SS wires were higher in the chlorhexidine than those in artificial saliva, but these differences were not statistically significant (*P* > 0.05). There is no comparative data on the frictional resistance between metal brackets and orthodontic archwires after immersion in a prophylactic chlorhexidine solution.

The present study AFM determined no significant difference in the average surface roughness of both wires before (as-received) and after immersion in either the chlorhexidine or the artificial saliva test environment. This finding may explain why chlorhexidine did not have significant influence on the frictional resistance between stainless steel brackets and the two types of evaluated orthodontic wires. In addition, the average surface roughness value for the as-received Ni-Ti was higher than that of SS wires. However, no significant difference in the frictional resistance between the two types of wires immersed in either of the test environments was observed (*P* > 0.05). It has been reported that the frictional forces of stainless steel archwires increased significantly in artificial saliva, and that of β-titanium archwire decreased when compared with the dry condition [4]. This might have been also the case for the stainless steel and nickel-titanium wires used in this study regardless of their surface roughness.

Prososki et al. [22] showed that stainless steel wires with the smoothest surface had higher frictional force values and suggested that there was no relation between surface roughness and coefficient of friction. Doshiand and Bhad-Patil [23] found no correlation between wire roughness and frictional resistance. These reports are consistent with the findings obtained in the present study. However, Nishio et al. [24] found that SS wire with the smoothest surface had the least frictional force values. Moreover, Saunders and Kusy [25] have suggested that the arch wire alloy, rather than bracket product type or surface roughness, may be more influential on the frictional characteristics.

Because frictional force is related to several factors, undesirable behavior in the frictional force values can be created. Therefore, it is difficult to compare studies, because of the different test methodologies and variables used, and these issues remain controversial in the literature.

The ligation between bracket and wire is another variable that influences the frictional force [24]. In the present study, the ligation method between the bracket and wire was standardized to eliminate this variable. However, large variation in the obtained data that was observed in this study could be due to the small sample size and employed test methodology. In addition, the minimal impact of chlorhexidine mouthrinse on the surface roughness or frictional resistance observed in this study might be due to the short-period immersion (1.5 h) of the stainless steel brackets and archwire assemblies in each test environment. The immersion period was chosen because chlorhexidine mouthrinses are usually prescribed for a short-term only between 4 to 12 weeks twice a day due to their adverse effects on tooth color and normal flora in the mouth. Chlorhexidine molecules would attach to the oral tissues and release gradually. Defining chlorhexidine concentration in the mouth after 30 s after expectoration is difficult. Thus, the 1.5-hchlorhexidine exposure in this study attempted to simulate 3-month accumulation of 1-min daily chlorhexidine mouthrinse applications. As with any *in vitro* study, the protocol cannot exactly simulate the real clinical situation.

It should be also noted that the frictional forces recorded in this study were substantially different from the actual applied forces in orthodontic movement. Many intraoral variables such as saliva, plaque, chewing, bone density, tooth number, anatomic configuration, and occlusion can influence frictional force levels, and were not evaluated in the present study. Another limitation of this study is that other types of orthodontic wires such as beta titanium wires have not been investigated, and thus, the obtained results cannot be extrapolated to them. Further clinical studies are required to investigate the effect of chlorhexidine-containing prophylactic agents during orthodontic treatments and on other types of archwires.

## Conclusions

Based on the results obtained from the present *in vitro* study, a 1.5-h immersion in the 0.2% chlorhexidine mouthrinse did not have a significant influence on archwires’ surface roughness or the frictional resistance between stainless steel brackets and the two types of orthodontic wires made of stainless steel and nickel-titanium alloys. Therefore, chlorhexidine-containing mouthrinses may be prescribed as non-destructive prophylactic agents on materials evaluated in this study for orthodontic patients.

## Competing interests

The authors declare that they have no competing interests.

## Authors’ contributions

THN participated in the design and coordination of the study. TH participated in the design of the study and drafted the manuscript. HF carried out the experimental work and participated in the sequence alignment. AM and ESER participated in the experimental work. All authors read and approved the final manuscript.
